# The Effect of Emergency Department Nurses’ Core Self‐Evaluations on Their Perceptions of Clinical Decision‐Making: A Cross‐Sectional Study

**DOI:** 10.1155/jonm/9948757

**Published:** 2026-02-13

**Authors:** Ya-Xin Song, Chang Lu, Ying An

**Affiliations:** ^1^ School of Nursing, Capital Medical University, Beijing, 100069, China, ccmu.edu.cn; ^2^ Nursing Department, Beijing Tongren Hospital, Capital Medical University, Beijing, 100730, China, ccmu.edu.cn

## Abstract

**Background:**

Nurses in emergency departments (EDs) typically face time and resource constraints when they make clinical decisions. Furthermore, core self‐evaluations, a fundamental, deep‐seated personality trait, represent a key factor influencing work motivation, job performance, and other behavioral outcomes. However, the relationship remains unclear between ED nurses’ core self‐evaluations and their perceptions of clinical decision‐making.

**Aim:**

This study aims to explore the impact of ED nurses’ core self‐evaluations on their perceptions of clinical decision‐making.

**Design:**

A cross‐sectional survey design was used.

**Methods:**

This cross‐sectional study surveyed 578 registered ED nurses from 30 public hospitals in Beijing, China. Data were collected via online and on‐site questionnaires, including sociodemographic characteristics, the Core Self‐Evaluations Scale, and the Clinical Decision‐Making in Nursing Scale. Statistical analyses included independent samples *t* tests, analysis of variance (ANOVA) with Welch’s correction for heterogeneity of variance, Pearson correlation analysis, and multiple linear regression analysis.

**Results:**

The ED nurses reported moderate levels of core self‐evaluations (39.16 ± 6.38) and relatively high perceptions of clinical decision‐making (150.12 ± 18.32). The statistically significant positive correlation was found between core self‐evaluations and perceptions of clinical decision‐making (*r* = 0.567, *p* < 0.001). Multiple linear regression indicated that head nurse/nurse manager position (*β* = 0.079, *p* = 0.049), perceived uncertainty (*β* = −0.085, *p* = 0.032), and dissatisfaction (*β* = −0.074, *p* = 0.019) with the ED working environment were significantly associated with clinical decision‐making perceptions.

**Conclusion:**

This study demonstrated that enhancing the core self‐evaluations of ED nurses may improve clinical decision‐making quality. Nursing managers should establish targeted incentive systems, provide relevant resource support, and encourage critical thinking and self‐reflection during training. These measures can strengthen ED nurses’ self‐efficacy and value recognition, ultimately advancing decision‐making competence and care quality.

## 1. Introduction

Overall patient volume in emergency departments (EDs) has increased rapidly, with most patients presenting with conditions characterized by rapid evolution, complexity, and criticality. Consequently, ED personnel require strong time management skills and the ability to engage in flexible clinical thinking and decision‐making [1–3]. Nursing is an integral component of healthcare [4]. Given their professional role, nurses maintain the closest contact with patients of all ED personnel throughout the entire course of medical treatment and are responsible for clinical decision‐making in nursing, ranging from patient assessment to evidence‐based care implementation [5].

Clinical decision‐making in nursing is defined as a core professional competency cultivated through clinical practice. This sophisticated process involves the dynamic integration of cognitive and behavioral dimensions, wherein nurses systematically synthesize theoretical knowledge with accumulated clinical expertise through critical reflection and analytical reasoning to formulate optimal decisions when confronted with complex clinical scenarios [6, 7]. The effectiveness of this decision‐making process is directly associated with care quality and, ultimately, patient health outcomes [8]. Phillips et al. [9] reported that clinical decision‐making relies on the cognitive guidance constructed through the integration of critical thinking, clinical reasoning, and clinical judgment, and that it ultimately facilitates the transformation from thinking to deciding whether to perform an action. Tiffen et al. [10] contend that clinical decision‐making constitutes the core competency of nurse practitioners (NPs). The authors formulated an NP‐specific definition and conceptual framework that characterized clinical decision‐making as a contextually bound, continuous, and evolving process wherein nurses systematically collect, interpret, and evaluate patient data to identify and select evidence‐based actions. Dual‐process theory has been widely recognized in cognitive and social psychology and posits the following two distinct systems of information processing: System 1 is rapid, automatic, and unconscious as it relies on experiential knowledge and contextual cues, whereas System 2 is characterized by slower, high effort that requires conscious engagement and that depends on rational cognitive analyses. When applied to the fields of judgment and decision‐making, these two systems correspond to intuition‐driven heuristic processing and reasoning‐based analytical processing [11]. In contrast, Hammond’s cognitive continuum theory (CCT) reconciles the traditional intuitive–analytical dichotomy by conceptualizing quasirationality, an intermediate region integrating both cognitive modes. The theory posits that judgment emerges from the interplay of task properties and cognitive processes [12]. A paradigmatic deconstruction of the decision‐making essence may enhance the multidimensional quality of clinical decision‐making in nursing, which would facilitate a shift toward task‐adapted precision, particularly in acute care contexts [5]. As nursing professionalization advances, nurses’ autonomy in clinical decision‐making has gained broader recognition in healthcare systems. Research demonstrates that nurses’ decision‐making is influenced by multilevel factors, including external constraints such as organizational culture and time pressure, as well as individual characteristics such as educational background, work experience, intuition, critical thinking, emotional intelligence, decision‐making styles, and nomophobia. Many of these factors largely resist objective quantification and comprehensive evaluation [5, 13–18]. This complexity is particularly pronounced in the ED, where clinical decision‐making is challenged by the convergence of a high‐stakes environment and a heterogeneous patient population. Compared with other hospital settings, ED nurses often care for patients with widely varying acuity and undifferentiated presentations. When making decisions, they must more thoroughly weigh multifaceted considerations [18–20].

The concept of core self‐evaluations, developed by Judge et al. [21], represents an individual’s fundamental self‐appraisal of abilities and values and encompasses four personality traits—self‐esteem, generalized self‐efficacy, neuroticism, and locus of control—which collectively underlie psychological processes and behavioral outcomes. Initially conceptualized in industrial–organizational psychology, core self‐evaluations explain and predict relationships between personality dispositions and work‐related behavioral variables such as job satisfaction and job performance [22]. Recent research extends investigations to domains such as job search behavior, career self‐efficacy, and learning engagement [23–25]. Research indicates that individuals with higher core self‐evaluations tend to appraise situations more positively, actively cope with challenges, and demonstrate stronger confidence in career‐related tasks, including information gathering, goal setting, and problem‐solving [26–28]. Therefore, higher core self‐evaluations may enhance an individual’s decision‐making confidence and proactive coping tendencies, thereby improving their decision‐making ability in complex situations.

However, within the nursing profession, the impact of core self‐evaluations, a higher‐order personality construct, on clinical decision‐making perceptions remains underexplored, with a particularly pronounced research gap among ED nurses who practice in highly uncertain environments. Therefore, this study aims to examine the relationship between core self‐evaluations and perceptions of clinical decision‐making in ED nurses. The findings are intended to provide an evidence‐based foundation for nursing managers to develop targeted training strategies, ultimately enhancing ED nurses’ professional efficacy and optimizing patient care quality.

## 2. Materials and Methods

### 2.1. Study Design and Participants

This multicenter, cross‐sectional study was conducted from October 2024 to January 2025 across 24 general hospitals and 6 specialty hospitals in Beijing, China. The ED nurses were recruited via convenience sampling through a combined online and on‐site survey approach. The inclusion criteria were as follows: registered ED nurses with a valid license, who are currently engaged in ED clinical practice, and who voluntarily participated in the study. The exclusion criteria were ED nurses from other hospitals and those not assigned clinical duties during the study period. The sample size was calculated on the basis of methodological guidelines from “*Medical Statistics, 5th edition,*” which recommend that the sample size should be 5–10 times the number of variables. In this study, 18 variables were included, comprising 17 general demographic variables and 1 independent variable associated with the scale dimensions. After an estimated 20% invalid response rate was considered, the minimum sample size was determined to be 108 participants. The research adhered to the Strengthening the Reporting of Observational Studies in Epidemiology guidelines.

### 2.2. Measurement

#### 2.2.1. Sociodemographic Information Questionnaire

Designed by the researchers through a systematic review of clinical decision‐making literature in nursing, the demographic questionnaire includes the following variables: gender, age, marital status, educational level, hospital level, hospital type, overall years of experience in the current hospital, years of experience in the ED, employment forms, professional title, position, rotating shifts, whether or not you have an emergency nursing specialist qualification, the number of critical care nursing training sessions attended in the past 3 years, whether or not you can effectively handle nurse–patient relationships, whether or not you have confidence in your competence for ED nursing practice, and whether or not you are satisfied with the ED working environment.

#### 2.2.2. Clinical Decision‐Making in Nursing Scale (CDMNS)

The CDMNS was originally developed by Jenkins [29] to assess nurses’ perceptions of clinical decision‐making, and the Chinese version of the CDMNS was translated and validated by He [30], who reported a Cronbach’s *α* coefficient of 0.78. This 40‐item scale comprises four dimensions: seeking information or new information, clarifying goals and values, seeking alternative actions, and evaluating or reevaluating outcomes. The items are rated on a five‐point Likert scale (1 = *never* to 5 = *always*), with 22 positively phrased items and 18 reverse‐scored items. Scores range from 40 to 200, where higher scores indicate perceptions of clinical decision‐making that are more positive. These scores could be categorized into three levels: 40.00–93.33, indicating minimal ability; 93.34–146.67, demonstrating intermediate ability; and 146.68–200.00, reflecting advanced clinical decision‐making ability. In this study, Cronbach’s *α* coefficient was 0.910.

#### 2.2.3. Core Self‐Evaluations Scale (CSES)

The CSES is used to measure individuals’ fundamental evaluations of their own abilities and values. Developed by Judge et al. [21], the scale incorporates four personality traits: self‐esteem, generalized self‐efficacy, neuroticism, and locus of control. Given the cross‐cultural variations in personality structures between Chinese and Western populations [31], we employed the culturally adapted Chinese version validated by Du et al. [32], who reported a Cronbach’s *α* coefficient of 0.830. This 10‐item scale is scored on a five‐point Likert scale (1 = *strongly disagree* to 5 = *strongly agree*), with higher total scores indicating higher levels of self‐confidence and self‐satisfaction. In the present study, Cronbach’s *α* coefficient was 0.905.

### 2.3. Data Collection and Quality Control

The data in this study were collected in two phases, combining online and offline approaches. In the first phase, questionnaires were distributed using the Question Star platform. Prior to the formal survey, the researchers provided training to the ED managers across the participating hospitals, who subsequently distributed the questionnaire links to the nursing staff via WeChat groups within their respective hospitals. The second phase involved the on‐site distribution of questionnaires to ED nurses on duty during morning‐shift handovers.

In both phases, the researchers explained the study objectives and emphasized adherence to the principles of anonymity and confidentiality to ensure response authenticity and reliability. Each questionnaire contained standardized instructions, detailed completion guidelines, and clear precautions. The participants were required to answer all of the questions before submission. A total of 721 questionnaires were collected. Following a rigorous data‐cleaning process, during which invalid questionnaires with abnormal response logic were excluded, 578 questionnaires were deemed valid for an effective response rate of 80.17%.

### 2.4. Statistical Analysis

The data were analyzed using IBM SPSS Statistics 27.0 software. The categorical variables were described as frequencies and percentages, whereas the continuous variables were expressed as the mean ± standard deviation (SD). The between‐group comparisons of two independent groups were analyzed using the independent samples *t*‐test, and differences among three or more groups were examined via a one‐way analysis of variance (ANOVA), applying Welch’s correction when heterogeneity of variance was present. Pearson correlation analysis and multiple linear regression analysis were applied to examine the relationships between demographic characteristics and core self‐evaluations with ED nurses’ perceptions of clinical decision‐making. Statistical significance was defined as a two‐sided *p* value < 0.05.

## 3. Results

### 3.1. Participant Characteristics

Among the 578 participants, the majority were female (81.8%). Their age ranged from 20 to 55 years, with a mean age of 31.11 years. With respect to marital status, 46.9% were single, and 52.1% were married. Bachelor’s degrees were held by 76.1%, whereas 24.9% held an emergency nursing specialty certification. Of the total, 84.1% worked day–night rotation shifts, whereas 15.9% worked permanently on the day shift. Further details are presented in Table 1.

**TABLE 1:** ED nurses’ demographics and differences in their perceptions of clinical decision‐making (*n* = 578).

Demographic variable	Frequency	Percentage (%)	Total CDMNS score *M* ± SD	*t/F*	*p*
Gender				0.002	0.999
Male	105	18.2	150.12 ± 1.76		
Female	473	81.8	150.12 ± 0.85		
Age				1.020	0.383
20∼25 years	137	23.7	150.58 ± 1.67		
26∼30 years	166	28.7	151.24 ± 1.43		
31∼35 years	139	24.0	147.80 ± 1.40		
≥ 36 years	136	23.5	150.66 ± 1.60		
Marital status				0.288	0.750
Single	271	46.9	150.39 ± 1.14		
Married	301	52.1	149.98 ± 1.04		
Divorced	6	1.0	144.83 ± 3.66		
Education level				1.280	0.279
Junior college and below	133	23.0	148.10 ± 1.70		
Undergraduate	440	76.1	150.66 ± 0.85		
Postgraduate and above	5	0.9	156.20 ± 10.74		
Hospital level				1.327	0.185
Tertiary	550	95.2	150.35 ± 0.78		
Secondary	28	4.8	145.64 ± 3.28		
Hospital type				−0.876	0.381
General hospital	516	89.3	149.89 ± 0.81		
Specialized hospital	62	10.7	152.05 ± 2.35		
Overall years of experience in the current hospital				0.770	0.464
≤ 5 years	203	35.1	151.40 ± 1.38		
6∼10 years	158	27.3	149.58 ± 1.37		
> 10 years	217	37.5	149.32 ± 1.21		
Years of experience in the emergency department				0.991	0.372
≤ 5 years	269	46.5	151.25 ± 1.17		
6∼10 years	139	24.0	148.80 ± 1.41		
> 10 years	170	29.4	149.42 ± 1.41		
Employment forms				0.566	0.572
Permanent position	166	28.7	150.80 ± 1.42		
Temporary position	412	71.3	149.85 ± 0.91		
Professional title				1.229	0.298
Nurse	156	27.0	149.96 ± 1.54		
Senior nurse	260	45.0	149.35 ± 1.11		
Supervisor nurse	152	26.3	150.95 ± 1.50		
Chief/associate chief nurse	10	1.7	160.00 ± 4.16		
Position				3.352	0.019
Frontline nurse	455	78.7	149.66 ± 0.86		
Preceptor	75	13.0	149.47 ± 2.03		
Clinical/teaching team leader	19	3.3	147.89 ± 4.59		
Head nurse/nurse manager	29	5.0	160.48 ± 3.02		
Rotating Shifts				3.361	< 0.001
Permanent day shift	92	15.9	155.96 ± 1.94		
Day–night rotation	486	84.1	149.02 ± 0.82		
Whether or not you have an emergency nursing specialist qualification?				−0.328	0.743
Yes	144	24.9	149.98 ± 0.88		
No	434	75.1	150.56 ± 1.55		
The number of critical care nursing training sessions attended in the past 3 years				−0.971	0.332
< 3 times	320	55.4	149.46 ± 1.05		
≥ 3 times	258	44.6	150.95 ± 1.10		
Whether or not you can effectively handle nurse‒patient relationships?				6.270	0.002
Yes	519	89.8	151.02 ± 0.80		
No	4	0.7	138.25 ± 10.30		
Uncertain	55	9.5	142.55 ± 2.34		
Whether or not you have confidence in your competence for emergency department nursing practice?				48.597^∗^	< 0.001
Yes	561	97.1	150.55 ± 0.77		
No	5	0.9	127.40 ± 2.25		
Uncertain	12	2.1	139.42 ± 2.74		
Whether or not you are satisfied with the emergency department working environment?				16.401	< 0.001
Yes	510	88.2	151.67 ± 0.80		
No	20	3.5	137.60 ± 3.05		
Uncertain	48	8.3	138.85 ± 2.29		

Abbreviations: *M*, mean; SD, standard deviation.

^∗^The Welch test was used because of unequal variances.

### 3.2. Common Method Bias Test

Given that all of the research data were derived from self‐reports by ED nurses, common method bias might arise. To address this issue, Harman’s single‐factor test was performed, incorporating all of the items from the demographic variables, the Nursing Clinical Decision‐Making Scale, and the CSES into an unrotated factor analysis. The results revealed 9 factors with eigenvalues greater than 1, collectively accounting for 59.705% of the total variance. Among them, the first factor explained 26.002% of the total, which is below the critical threshold of 40%. These findings indicate that there was no significant common method bias in this study.

### 3.3. Perceptions of Clinical Decision‐Making Across Different Demographic Characteristics

The results of this study revealed significant differences in perceptions of clinical decision‐making based on factors such as position, rotating shifts, ability to effectively handle nurse–patient relationships, confidence in ED nursing work competence, and satisfaction with the ED working environment (all *p* < 0.05) (Table 1).

### 3.4. Scale Scores and Correlation Analysis

The study data indicated that ED nurses’ perceptions of clinical decision‐making were relatively high, with a mean score of 150.12 ± 18.32. Among the subscales, seeking information or new information yielded the lowest score of 35.81 ± 4.91, whereas evaluating or reevaluating outcomes achieved the highest score of 39.31 ± 5.55. Additionally, the average core self‐evaluations score was 39.16 ± 6.38, indicating a moderate level (Table 2).

**TABLE 2:** Descriptive statistics and correlation analysis for CDMNS and CSES (*n* = 578).

Variables	*M* ± SD	1	2	3	4	5	6
1. Clinical decision‐making in nursing	150.12 ± 18.32	1					
2. Seeking information or new information	35.81 ± 4.91	0.871^∗∗^	1				
3. Clarifying goals and values	37.72 ± 5.11	0.885^∗∗^	0.674^∗∗^	1			
4. Seeking alternative actions	37.27 ± 4.88	0.911^∗∗^	0.738^∗∗^	0.745^∗∗^	1		
5. Evaluating or reevaluating outcomes	39.31 ± 5.55	0.916^∗∗^	0.723^∗∗^	0.751^∗∗^	0.790^∗∗^	1	
6. Core self‐evaluations	39.16 ± 6.38	0.567^∗∗^	0.447^∗∗^	0.514^∗∗^	0.560^∗∗^	0.510^∗∗^	1

Abbreviations: CDMNS, Clinical Decision‐Making in Nursing Scale; CSES, Core Self‐Evaluations Scale.

^∗∗^ *p* < 0.001.

The Pearson correlation analysis revealed that core self‐evaluations were significantly positively correlated with perceptions of clinical decision‐making and its four subscales (*r* = 0.563, *p* < 0.001). These findings indicate that higher core self‐evaluations are associated with enhanced perceptions of clinical decision‐making (Table 2).

### 3.5. Multiple Linear Regression Analysis of CDMNS

A multiple linear regression analysis was employed to examine the influence of demographic characteristics and core self‐evaluations on the perceptions of clinical decision‐making among ED nurses, with no significant multicollinearity detected. The regression model indicated that core self‐evaluations (*β* = 0.542, *p* < 0.001) and head nurse/nurse manager position (*β* = 0.079, *p* = 0.049) were significant positive predictors. Conversely, perceived uncertainty (*β* = −0.085, *p* = 0.032) and dissatisfaction (*β* = −0.074, *p* = 0.019) with the ED working environment were significant negative predictors (Table 3).

**TABLE 3:** Results of the multiple linear regression analysis for predicting ED nurses’ perceptions of clinical decision‐making (*n* = 578).

Variable	*B*	SE	*β*	*t*	*p*	95% CI	
Constant	91.814	4.766		19.265	< 0.001^∗∗^	82.454	101.175
Position (ref. frontline nurse)							
Preceptor	−1.950	1.868	−0.036	−1.044	0.297	−5.619	1.719
Clinical/teaching team leader	−0.223	3.570	−0.002	−0.062	0.950	−7.234	6.789
Head nurse/nurse manager	6.608	3.362	0.079	1.966	0.049^∗^	0.005	13.211
Rotating Shifts (ref. permanent day shift)							
Day–night rotation	−2.505	2.026	−0.050	−1.236	0.217	−6.485	1.475
Whether or not you can effectively handle nurse‒patient relationships? (ref. yes)							
No	−2.330	7.712	−0.011	−0.302	0.763	−17.478	12.817
Uncertain	1.419	2.293	0.023	0.619	0.536	−3.084	5.922
Whether or not you have confidence in your competence for emergency department nursing practice? (ref. yes)							
No	−10.641	7.013	−0.054	−1.517	0.130	−24.415	3.133
Uncertain	4.480	4.598	0.035	0.974	0.330	−4.551	13.511
Whether or not you are satisfied with the emergency department working environment? (ref. yes)							
No	−7.442	3.470	−0.074	−2.145	0.032^∗^	−14.258	–0.627
Uncertain	−5.608	2.383	−0.085	−2.353	0.019^∗^	−10.289	–0.927
CSES	1.556	0.104	0.542	14.965	< 0.001^∗∗^	1.352	1.761

*Note: B*, unstandardized regression coefficient; *β*, standardized regression coefficient; 95% CI, 95% confidence interval; *R* = 0.592; *R* ^2^ = 0.351; adjusted *R* ^2^ = 0.338; *F* = 27.796.

Abbreviation: SE, standard error.

^∗^ *p* < 0.05.

^∗∗^ *p* < 0.001.

## 4. Discussion

This study explored the relationship between core self‐evaluations and perceptions of clinical decision‐making among ED nurses in Beijing, China. The results indicate that ED nurses’ core self‐evaluations significantly influence their clinical decision‐making perceptions. Therefore, these findings highlight the critical importance of fostering self‐affirmation in terms of abilities and values in practice, as doing so may improve nurses’ perceptions of their clinical decision‐making capabilities.

The findings revealed that the participants’ core self‐evaluations were at an overall moderate level. Notably, these scores were slightly higher than those reported in several other studies conducted in China [24, 33, 34], with this discrepancy potentially attributable to factors such as participants’ work units and sample sizes. As a frontline setting for managing critically ill patients in hospitals, EDs routinely encounter patient overcrowding [35]. To ensure safety for patients with clinically urgent and complex conditions, ED nurses must demonstrate exceptional clinical judgment and critical thinking competencies [17]. These professional requirements may consequently cultivate heightened self‐evaluations. Additionally, prior research indicates a positive correlation between nurses’ age and their sense of personal accomplishment and suggests that years of clinical experience significantly influence the development of their core competencies [36, 37]. In this study, 53.4% of the participants had 6 years or more of clinical experience. Such experience may enable ED nurses to progressively master professional roles, thereby cultivating greater occupational efficacy and self‐actualization–driven values through accumulated expertise [38].

In this study, the mean score for ED nurses’ perceptions of clinical decision‐making was at a relatively high level. This result is lower than that reported by Zhang et al. [16] and Jawabreh [39] but slightly higher than those in other studies [14, 17, 40]. Such cross‐study discrepancies may stem from factors such as variations in educational backgrounds, training systems, and organizational cultures. At present, models of medical training and curricular frameworks differ across countries. For instance, many countries have implemented residency programs and incorporated curricula explicitly designed to cultivate critical thinking skills [41]. In contrast, hierarchical structures and standardized management systems within China’s medical training infrastructure require further refinement [42, 43]. Furthermore, this phenomenon can be explained from a dual perspective. First, the distinctive operational model of EDs—characterized by acute patient conditions, diagnostic complexity, and high‐intensity workloads—requires nurses to continuously refine systematic theoretical knowledge and resuscitation skills, thereby enhancing dynamic decision‐making capacity. Second, as demonstrated by Sharma et al. [44] in their study on nurse roles in patient flow management, external constraints, particularly resource allocation challenges and time pressures, may substantially limit the full utilization of ED nurses’ decision‐making capabilities in clinical practice. Moreover, the scores across the four dimensions of nursing clinical decision‐making perceptions were consistent with the patterns observed in other studies of nurse populations in China [14, 17, 45]. The high score in the evaluation or reevaluation of outcomes may stem from the fact that ED nurses operate under high‐pressure conditions, often making decisions due to time constraints or external pressures while facing accountability dilemmas [46]. Consequently, their decision‐making requires balancing patients’ rights and safety against multifaceted clinical factors, which may lead them to place greater emphasis on the quality of their decisions. The low score for seeking information or new information may indicate a need to enhance this competency among Chinese ED nurses. As Dresser et al. [47] reported, nurses’ clinical judgments during patient deterioration involve complex, multidimensional processes in which information collection directly determines the scientific rigor of decision‐making [45]. These findings underscore the necessity for institutional support to strengthen ED nurses’ abilities in collecting and integrating new information, enabling timely identification of critical nursing issues.

The results indicated that the position of head nurse or nurse manager significantly influenced the perceptions of clinical decision‐making. This finding may be explained by two main reasons. First, individuals in these roles typically possess extensive clinical experience, equipping them with substantial professional knowledge and practical skills. Their responsibilities encompass supervising all aspects of nursing care and enhancing staff work engagement and satisfaction, which likely fosters greater decision‐making confidence and professional competence [48, 49]. Second, variations in research findings may also stem from contextual factors in the decision‐making process, such as task complexity, information availability, and the influence of cognitive biases like overconfidence [50, 51]. Meanwhile, this study found that ED nurses’ satisfaction with their work environment significantly influenced their perceived clinical decision‐making ability. Previous research indicates that a positive work environment can enhance both the quality of nurses’ clinical decisions and their productivity [52]. However, ED nurses are consistently exposed to multiple occupational stressors. In our study, 84.1% of participants worked rotating day and night shifts. Such conditions can adversely affect their physical and mental health, reduce job satisfaction, and impair cognitive and executive function [53–56].

Previous studies have demonstrated that effective clinical decision‐making in nursing depends on key factors, including clinical competence, nurse–patient relationship management, and self‐efficacy [57, 58]. Our findings corroborate this relationship, suggesting that nurses’ self‐affirmation of abilities and values may act as an intrinsic motivator, optimizing the formulation and implementation of clinical decisions.

As a fundamental motivational trait, core self‐evaluations have been linked to multidimensional factors, including job performance, attitudes, and behaviors [33, 38, 59]. Nurses who exhibit elevated core self‐evaluations are more likely to demonstrate increased work engagement and innovative thinking. Furthermore, Saei et al. [60] delineated dual functions through which core self‐evaluations modulate the emotional labor–burnout relationship among nurses. First, core self‐evaluations function as a mediating resource that facilitates deep acting, such as adjusting genuine emotions to align with professional requirements, thereby enhancing personal accomplishment. Second, core self‐evaluations serve as a regulatory buffer that mitigates the adverse outcomes of surface acting, such as emotional faking, consequently reducing burnout susceptibility. This framework aligns with that of Geuens et al. [36], who demonstrated that core self‐evaluations directly govern emotional exhaustion during burnout progression. Collectively, these findings underscore the critical role of personal traits in alleviating work stress and optimizing care quality. However, it is noteworthy that cultural background shapes individuals’ self‐perception, values, and social norms. For instance, individualistic cultures emphasize self‐actualization and tend to employ analytical cognition. In contrast, collectivist cultures, such as China’s, prioritize a sense of belonging to the group and favor holistic cognition. In such contexts, an individual’s self‐concept is often more closely intertwined with social relationships and role obligations. Consequently, the decision‐making process may prioritize consensus‐building through team communication and careful deliberation over reliance solely on independent judgment [61–64]. Given that cultural influences are typically complex and multifaceted, further cross‐cultural studies are warranted to explore this issue in depth.

Clinical decision‐making is a cornerstone of nursing practice and directly influences care quality. As a key personality construct for predicting nurses’ work‐related behaviors, core self‐evaluations are significantly correlated with clinical decision‐making competence. Farčić et al. [65] reported that nurses with higher core self‐evaluations demonstrate greater professional confidence, stronger independent decision‐making tendencies, and stronger conviction in managing diverse workplace scenarios. However, research gaps persist regarding the relationship between core self‐evaluations and clinical decision‐making perceptions among Chinese ED nurses. Our findings align with those of Farčić et al. [65] to reveal that core self‐evaluations shape ED nurses’ work‐related behaviors and decision‐making perceptions in clinical practice.

This study has significant implications for nursing management. EDs are currently facing sustained workload overload and heightened occupational hazards, which precipitate pervasive burnout, compromised job satisfaction, and increased turnover intentions among staff [66]. These challenges collectively constrain professional development and the optimization of care quality. Consequently, in training programs, nursing managers should prioritize the cultivation of core self‐evaluations among ED nurses, refine incentive systems, and employ resource‐empowerment strategies to enhance these self‐evaluations, thereby improving the quality of nursing decision‐making.

First, nursing managers should provide adequate psychological resource support, foster a conducive work environment, and guide nurses to cultivate a positive mindset. This approach intrinsically enhances core self‐evaluations, thereby improving perceived job competence and optimizing decision‐making quality.

Concurrently, nursing managers should promote critical reflection on nurses’ overall self‐assessments to mitigate cognitive biases arising from overconfidence in clinical practice [51, 65, 67]. Such reflective practice addresses implementation gaps in decision‐making processes and facilitates evidence‐based, precise patient assessments and scientific decision‐making plans.

Finally, novice and experienced nurses exhibit distinct patterns in clinical judgment and decision‐making, attributable to differences in experience and knowledge [5, 68]. As emphasized by Zhao et al. [69], new employees often face pressures such as information scarcity and role ambiguity during organizational socialization. Supervisory developmental feedback—defined as actionable information provided by managers to facilitate employee learning, development, and work improvement—enhances newcomers’ organizational identification by serving as a critical information resource [70, 71]. This process enhances task performance and work initiative. Moreover, individuals with high core self‐evaluations tend to amplify these positive effects [69]. Therefore, we recommend that nursing managers adopt differentiated development strategies for ED nurses, tailored to variations in their core self‐evaluations and seniority. With respect to nurses with lower seniority and core self‐evaluations, nursing managers should prioritize providing resources that facilitate rapid adaptation to emergency roles, along with intensified performance guidance and supervisory attention. This support is crucial for catalyzing their transition into an “organizational insider” role, ultimately fostering professional identity and self‐affirmation. For nurses with higher seniority and core self‐evaluations, nursing managers should focus on maximizing empowerment within appropriate boundaries and guiding employees to leverage their existing resources and exercise autonomous initiative. This approach aims to unlock their professional potential, thereby enhancing care quality and ensuring patient safety.

### 4.1. Limitations

This study has several limitations. First, the participants were recruited from a relatively small‐scale sample, and the cultural differences between Eastern and Western contexts may limit the generalizability of the findings. Future research should validate these findings in more culturally diverse populations. Moreover, this study employed self‐reported scales, which may be subject to social desirability bias. Additionally, due to the high‐pressure work environment in the ED, participants’ responses could have been influenced by confounding factors such as psychological distress and excessive workload during data collection, potentially compromising the accuracy of self‐assessed core self‐evaluations and decision‐making confidence. Future studies should adopt longitudinal or intervention‐based designs, incorporating objective measures of such confounders, to better elucidate the causal relationship. Finally, while this study revealed an association between core self‐evaluations and perceptions of clinical decision‐making among ED nurses, the potential moderating role of factors such as decision‐making styles in this relationship warrants in‐depth exploration. Such exploration would enrich the research conclusions and provide more targeted practical insights for building the capacity of clinical nursing staff.

## 5. Conclusion

This study examined the association between core self‐evaluations and perceptions of clinical decision‐making among ED nurses. We found that higher core self‐evaluations were significantly associated with more positive perceptions of clinical decision‐making, highlighting the critical need to foster the development of core self‐evaluations in high‐acuity clinical environments. Therefore, nursing managers should enhance resource empowerment within training programs and encourage nurses to consistently engage in critical thinking and reflective practices. Additionally, managers should implement hierarchical training strategies tailored to nurses’ core self‐evaluations and work experience, with the aim of enhancing their work engagement and clinical decision‐making quality. Future research should employ longitudinal or intervention‐based designs that incorporate real‐world clinical data to better establish causality. Furthermore, the potential mediating role of factors such as decision‐making styles in this relationship should be explored through a mediation analysis.

## Author Contributions

Ya‐Xin Song: writing–original draft, writing–review and editing, visualization, methodology, investigation, formal analysis, and data curation.

Chang Lu: visualization and data curation.

Ying An: writing–review and editing, visualization, supervision, validation, methodology, investigation, and data curation.

## Funding

This research did not receive any designated funding.

## Ethics Statement

This study was approved by the Institutional Review Board of Beijing Tongren Hospital, Capital Medical University, with the ethical approval number (TREC2024‐KY111). The study was conducted in strict adherence to the World Medical Association Declaration of Helsinki. Prior to the initiation of the study, all participants were fully informed and voluntarily consented to participate. Confidentiality was rigorously maintained, and participants’ privacy rights were protected throughout the study.

## Conflicts of Interest

The authors declare no conflicts of interest.

## Data Availability

The data that support the findings of this study are available from the corresponding author upon reasonable request.
